# Characterizing Physical Activity Trajectories Preceding Incident Major Depressive Disorder Diagnosis With Consumer Wearable Devices in the All of Us Research Program: Retrospective Nested Case-Control Study

**DOI:** 10.2196/93164

**Published:** 2026-05-04

**Authors:** Yuezhou Zhang, Amos A Folarin, Hugh Logan Ellis, Rongrong Zhong, Callum Stewart, Heet Sankesara, Hyunju Kim, Shaoxiong Sun, Abhishek Pratap, Richard JB Dobson

**Affiliations:** 1 Department of Biostatistics & Health Informatics, Institute of Psychiatry, Psychology and Neuroscience, King's College London, SGDP Centre, IoPPN, De Crespigny Park, Denmark Hill London, London, United Kingdom, 44 2078480473; 2 Institute of Health Informatics, University College London, London, United Kingdom; 3 NIHR Biomedical Research Centre at South London and Maudsley, NHS Foundation Trust, London, United Kingdom; 4 NIHR Biomedical Research Centre at University College London Hospitals, NHS Foundation Trust, London, United Kingdom; 5 Health Data Research UK, University College London, London, United Kingdom; 6 Clinical Research Center & Division of Mood Disorders, Shanghai Mental Health Center, Shanghai Jiao Tong University School of Medicine, Shanghai, China; 7 Department of Computer Science, University of Sheffield, Sheffield, United Kingdom; 8 Boehringer Ingelheim Pharmaceuticals, Inc., Ridgefield, CT, United States; 9 Department of Biomedical and Health Informatics, University of Washington, Seattle, WA, United States

**Keywords:** major depressive disorder, depression, physical activity, wearable devices, mobile health, mHealth, longitudinal studies, case-control studies, electronic health records

## Abstract

**Background:**

Low physical activity (PA) is a well-established risk factor for major depressive disorder (MDD). However, the temporal dynamics of PA preceding an incident clinical diagnosis of MDD remain poorly characterized, particularly using long-term, objective measures collected in real-world settings.

**Objective:**

This study aimed to characterize trajectories of wearable-measured PA during the year preceding incident MDD diagnosis and identify the timing of within-person changes.

**Methods:**

We conducted a retrospective nested case-control study using linked electronic health record and wearable (Fitbit) data from the All of Us Research Program. Adults with at least 6 months of valid Fitbit PA data in the 12 months preceding diagnosis were included. Incident MDD cases were identified based on a first electronic health record–recorded diagnosis and matched to MDD-free controls on age, sex, BMI, and calendar time of diagnosis, with up to 4 controls per case. Daily steps and moderate to vigorous PA (MVPA) were aggregated into monthly averages. Linear mixed-effects models were used to compare prediagnostic PA trajectories between cases and controls over a retrospective time scale from −12 to 0 months. Among cases, within-person contrasts were used to identify when PA levels first showed statistically significant deviations relative to levels observed 12 months before diagnosis. Exploratory analyses assessed heterogeneity by demographic factors.

**Results:**

The analytic cohort included 4104 participants (n=829, 20.2% incident MDD cases and n=3275, 79.8% matched controls; n=3355, 81.7% women; median age 48.4, IQR 36.3-61.3 years). Compared with controls, individuals who developed MDD exhibited consistently lower overall PA and significant downward trajectories in both daily steps and MVPA during the year preceding diagnosis (global trajectory tests; *P*<.001 for both outcomes). Differences widened progressively over time, indicating accelerating declines as diagnosis approached. Among cases, statistically significant changes in daily step counts emerged approximately 4 months before diagnosis (−145, 95% CI −253 to −37 steps vs month −12; *P*=.02) and reached −428 (95% CI −531 to −326) steps at diagnosis (*P*<.001). Declines in MVPA emerged approximately 5 months before diagnosis (−2.48, 95% CI −4.32 to −0.64 minutes; *P*=.02) and reached −5.61 (95% CI −7.35 to −3.86) minutes at diagnosis (*P*<.001). Furthermore, exploratory analyses suggested heterogeneity in prediagnostic trajectories across demographic subgroups, including steeper declines among men, more pronounced reductions in activity intensity at older ages, and persistently lower activity levels with flatter trajectories among individuals with obesity.

**Conclusions:**

Unlike prior studies lacking objective PA assessment before MDD diagnosis, this study linked wearable and clinical data to characterize long-term prediagnostic trajectories in real-world settings. We observed sustained within-person declines emerging 4 to 5 months before diagnosis, providing insights into temporal dynamics preceding clinical recognition. These findings suggest that wearable-based monitoring may offer scalable early signals for risk stratification, prevention, and intervention for MDD.

## Introduction

Major depressive disorder (MDD) is a leading cause of disability worldwide [1] and is associated with substantial adverse outcomes, including premature mortality [2], functional impairment [3], increased medical comorbidity [4], and suicide [5]. Given its substantial individual and societal burden [1], early identification of MDD is critical. Preventive strategies [6] and early interventions [7] may be most effective during the prodromal or initial stages. However, symptoms of MDD in the prodromal or early stages are often difficult to detect in routine clinical practice, contributing to delays in diagnosis [8,9]. This highlights the need for objective and scalable approaches to characterize changes preceding clinical recognition of MDD.

Low physical activity (PA) has been identified as a risk factor for MDD in prospective cohort studies and meta-analyses [10-12], with longitudinal evidence indicating that reductions in PA may precede worsening depressive symptoms [13]. These patterns may reflect the underlying biological and behavioral processes implicated in MDD, including alterations in psychomotor, inflammatory, and neuroendocrine functioning [14-16]. In this context, changes in PA may precede clinical recognition of MDD. However, much of the large-scale epidemiological existing evidence relies on self-reported PA, which is subject to recall bias [11], or on accelerometer-based assessments conducted over short durations or at infrequent follow-up time points [17]. Consequently, current studies provide limited insights into the timing and pattern of PA changes preceding a clinical MDD diagnosis. In particular, it remains unclear when PA begins to deviate from an individual’s prior level and whether these changes vary across population subgroups. Addressing these questions requires longitudinal, objective measures of PA collected in real-world settings. Linking these data to clinical diagnostic information enables characterization of behavior changes prior to diagnosis as illness unfolds in routine care.

Advances in sensor technology and the widespread adoption of consumer wearable devices have enabled passive, continuous monitoring of real-world PA at scale. Devices such as Fitbit trackers capture daily step counts and activity intensity over extended periods with minimal user burden [18,19], representing key correlates of functioning and psychomotor activity [14]. Recent mobile health studies have demonstrated negative associations between objectively measured PA and depression severity [20-23], suggesting that such measures may complement symptom-based assessments by informing risk stratification and prompting earlier clinical evaluation. Nevertheless, prior mobile health studies have been limited by modest sample sizes, relatively short monitoring durations, or the absence of linkage to clinical diagnoses [20]. As a result, the temporal dynamics of objectively measured PA prior to clinical MDD diagnosis remain insufficiently characterized.

Few large-scale datasets integrate long-term wearable data with electronic health records (EHRs) in a manner that enables examination of prediagnostic PA trajectories and subgroup heterogeneity. The All of Us Research Program (AoURP) provides such an integrated resource, comprising a large, ongoing national cohort in which participants consent to share EHRs, health surveys, biospecimens, physical measurements, and wearable data [24]. By linking historical and prospective Fitbit data with EHR-based diagnoses, this study enables examination of long-term PA trajectories preceding clinical MDD diagnosis. The primary aim of this study was to characterize trajectories of wearable-measured PA during the 12 months preceding incident MDD diagnosis and identify the timing of within-person deviations from prior habitual PA levels. The secondary aim was to assess whether the patterns of prediagnostic PA changes vary across population subgroups.

## Methods

### Data Source

We used data from the AoURP, an ongoing national longitudinal cohort funded by the US National Institutes of Health [24] with the long-term goal of enrolling at least 1 million participants. The study design and data collection procedures have been described previously [24,25].

This analysis used the controlled tier dataset, version 8 (C2024Q3R8), including participants enrolled between May 2017 and October 2023. Participant demographics and baseline data were collected during the digital enrollment. For participants who consented to share EHR and Fitbit data, their historical (pre-enrollment) EHR and Fitbit data were made available through their participating health care provider organizations and linked Google Fitbit accounts, respectively [24-26]. In this data version, 36,614 individuals had linked EHR and Fitbit data available.

### Ethical Considerations

This study was a secondary analysis of deidentified data obtained from the AoURP and did not involve direct interaction with human participants. In accordance with applicable regulations, this work was determined to be exempt from additional institutional review board review as it used existing, deidentified research data. Access to deidentified data was restricted to authorized study investigators who completed required All of Us Responsible Conduct of Research training, and all analyses were conducted within the secure, cloud-based Researcher Workbench environment. In accordance with the AoURP Data and Statistics Dissemination Policy [27] analytic results for groups with fewer than 20 participants were not reported to minimize the risk of participant reidentification.

All participants in the AoURP provided informed consent at enrollment, including consent for future secondary analyses of their data. Data privacy and confidentiality were protected through multiple safeguards, including secure cloud-based data storage, restricted access to deidentified data, and mandatory confidentiality and data use agreements. Participant compensation of US $25 for the collection of biological specimens (eg, blood, saliva, or urine) was provided by the AoURP in the form of cash payments, gift cards, or electronic vouchers as applicable. No images or figures included in the manuscript or supplementary materials contain information that could identify individual participants.

### Measures and Covariates

#### Fitbit PA Data

PA in this study was quantified using daily step counts and moderate to vigorous PA (MVPA), which intuitively capture overall activity volume [28] and the activity intensity emphasized in established public health guidelines [29], respectively. Fitbit classifies activity intensity into device-defined categories using metabolic equivalent of task–based criteria, consistent with intensity frameworks widely adopted in research-grade accelerometry [30,31]. In this study, daily MVPA (minutes per day) was calculated as the sum of device-labeled “fairly active” minutes plus twice the number of “very active” minutes, consistent with established definitions [32].

To ensure data quality, only days with at least 10 hours of wear time and daily step counts between 100 and 45,000 were considered valid following prior AoURP studies [33-35]. Monthly averages of daily step counts and MVPA minutes were calculated to reduce day-to-day variability related to missingness and short-term fluctuations, consistent with prior AoURP analyses [33-35]. Months with more than 10 valid days were considered valid and retained for analysis [35].

#### Incident MDD Definition

Following definitions used in prior AoURP research [33,36], incident MDD cases were defined as participants whose first recorded MDD diagnosis occurred during their Fitbit monitoring period, with no prior MDD diagnosis. Diagnoses were identified from EHR data using standardized concept identifiers from the Observational Medical Outcomes Partnership Common Data Model, which harmonize diagnoses across vocabularies including the Systematized Nomenclature of Medicine; *International Classification of Diseases, Ninth Revision, Clinical Modification*; and *International Classification of Diseases, Tenth Revision, Clinical Modification* [37].

#### Covariates

Age, sex, and BMI were included as covariates given their established associations with both PA and risk of MDD [38].

### Inclusion and Exclusion

Participants were required to be aged 19 years or older at the time of diagnosis to ensure that they were adults (≥18 years) throughout the 1-year prediagnostic observation period. In addition, individuals with any recorded diagnosis of bipolar disorder, schizophrenia, or schizoaffective disorder prior to the diagnosis date were excluded. The Observational Medical Outcomes Partnership concept identifiers used for inclusion and exclusion, along with their corresponding *International Classification of Diseases, Ninth Revision, Clinical Modification*, and *International Classification of Diseases, Tenth Revision, Clinical Modification*, codes, are provided in Table S1 in Multimedia Appendix 1. To ensure sufficient prediagnostic wearable data, cases were required to have at least 6 valid months of Fitbit PA data within the 12 months preceding the diagnosis month.

### Sampling and Matching Procedures

A nested case-control design [39-41] was used to construct the comparison group. Cases and controls were matched at the diagnosis month (hereafter referred to as the matching month) on age (within 1 year), sex, and BMI category, which were selected a priori given their strong associations with both PA and depression risk. BMI was defined using the measurement closest to 1 year before the matching month to minimize potential influence of emerging MDD on body weight. Eligible controls were selected from the risk set of participants who were aged 19 years or older at the matching month and had no recorded MDD diagnosis. Under this risk set sampling design, controls were not required to remain free of MDD during subsequent follow-up, consistent with standard nested case-control methodology [39-41]. Participants with a prior diagnosis of bipolar disorder, schizophrenia, or schizoaffective disorder were excluded. Controls were also required to meet the same Fitbit PA data availability criteria as cases. For each case, up to 4 controls were selected, a ratio chosen to improve statistical efficiency while maintaining matching quality and consistency with prior trajectory-based nested case-control studies [39,40]. When more than 4 eligible controls were available, controls were randomly sampled without replacement within or between cases to avoid repeated use of the same individuals and to simplify the correlation structure of the longitudinal analyses [40]. Additional sociodemographic factors were not included in matching to avoid overmatching and preserve statistical efficiency.

### Statistical Analysis

Linear mixed-effects models with participant-specific random intercepts were used to estimate PA trajectories over time, allowing for the inclusion of all available observations from eligible participants and accommodating missing data under a missing at random assumption. Formal testing of the missing completely at random assumption was not informative as missingness patterns were highly correlated across PA variables (eg, step count and MVPA were often missing concurrently). A retrospective time scale from −12 to 0 months was defined, with time 0 corresponding to the diagnosis month for cases and the matching month for controls. Models included case-control status (coded as 0 for controls and 1 for cases), linear and quadratic time terms (time and time squared), and their interaction terms, as well as matching variables. Quadratic terms were included to allow for potential nonlinear temporal patterns. Differences in trajectories between cases and controls were evaluated using time × case and time squared × case interaction terms, with a joint Wald test used to evaluate whether overall temporal patterns differed by case-control status. Model-derived marginal means were used to estimate and compare monthly trajectories between cases and controls. The statistical significance of between-group differences at each month was assessed using contrast *P* values, with *P* values adjusted for multiple comparisons using the Benjamini-Hochberg procedure [42].

Among individuals with incident MDD, marginal means were estimated for each month, and pairwise contrasts comparing each month with month −12 were used to identify when PA began to differ relative to the earliest month. To assess potential heterogeneity in prediagnostic PA trajectories, exploratory case-only models were extended to include interactions between time variables and subgroup indicators for sex, age, and BMI.

### Sensitivity Analyses

To assess the robustness of our findings, we conducted prespecified sensitivity analyses. First, to evaluate whether device wear compliance differed between cases and controls, we examined trajectories of monthly valid Fitbit wear days over time. Linear mixed-effects models including time, group (case and controls), and their interaction were used to assess whether wear compliance declined differentially as diagnosis approached.

Second, to reduce potential confounding from intercurrent medical illness or injury, we excluded participants with newly recorded incident diagnoses of conditions known to affect PA within 1 year prior to diagnosis (or the matching month for controls), including musculoskeletal disorders (eg, fractures of the bone), acute cardiovascular events (eg, myocardial infarction, stroke, transient ischemic attack, and heart failure), and cancer (diagnostic codes are provided in Table S2 in Multimedia Appendix 1) and repeated the analyses in the restricted cohort.

Third, because incident MDD was defined based on the absence of prior EHR-documented diagnoses, the extent of available EHR history is an important consideration. Therefore, we quantified the duration of available EHR history prior to diagnosis (or matching month for controls), defined as the time between the first recorded EHR activity (eg, laboratory result, diagnosis, procedure, visit, or vital sign entry) and the MDD diagnosis date. To assess the robustness of our findings, we conducted sensitivity analyses restricted to participants (both cases and controls) with at least 5 and 10 years of available EHR history.

## Results

### Participant Characteristics

**Figure 1.**
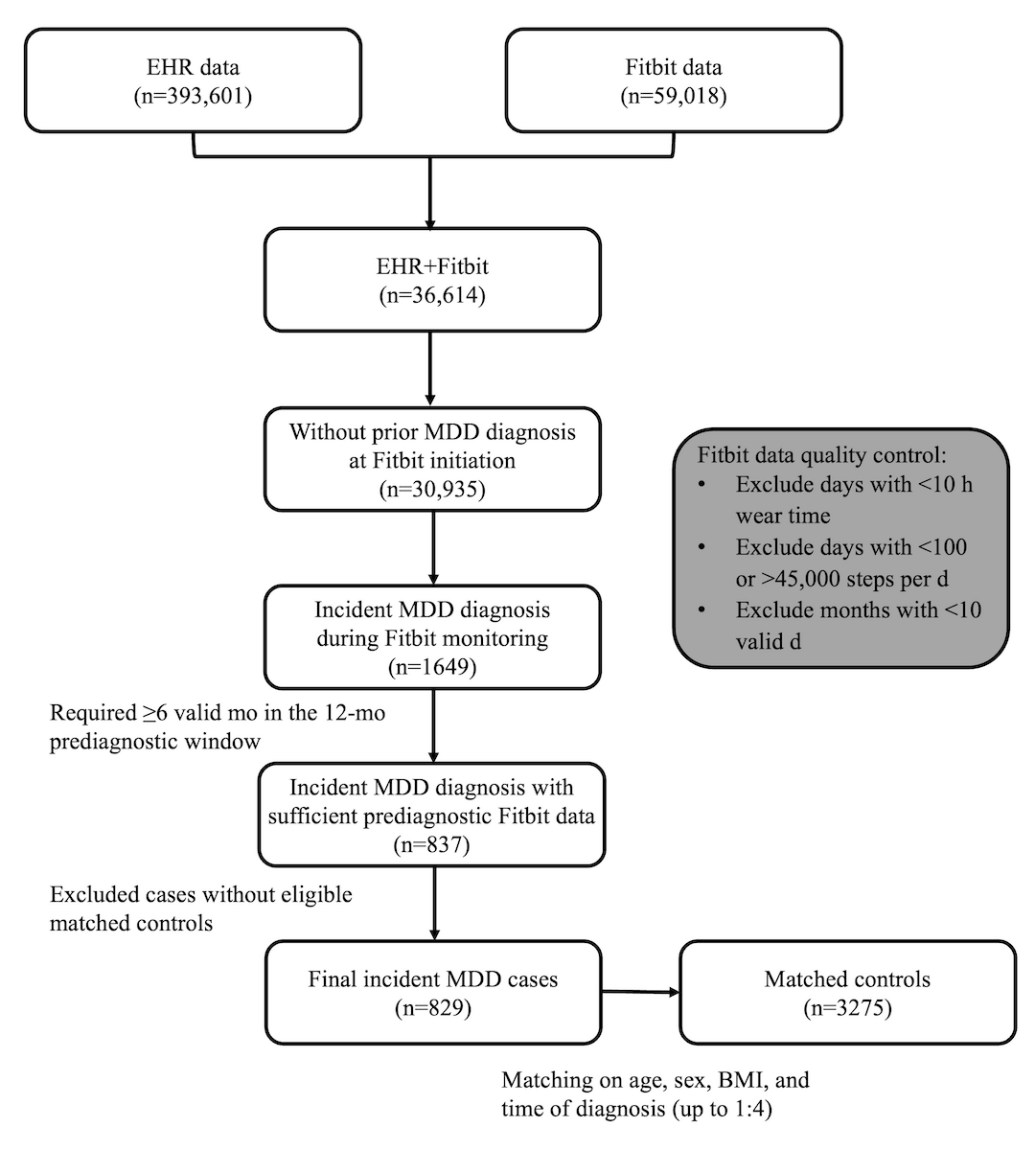
Flowchart of participant selection and cohort construction for incident major depressive disorder (MDD) cases and matched controls in a nested case-control study using linked electronic health record (EHR) and Fitbit data from the All of Us Research Program.

This study included 4104 participants, comprising 829 (20.2%) individuals with incident MDD and 3275 (79.8%) matched controls. Participant selection and cohort construction are shown in Figure 1. The median age of the overall cohort was 48.4 (IQR 36.3‐61.3) years; 81.7% (n=3355) of the participants were women, and 82.5% (n=3384) were White individuals. Cases and controls had similar distributions of age, sex, BMI, race, and ethnicity, with all standardized mean differences below 0.1, indicating adequate balance between groups (Table 1). Included participants had a mean of 11.05 (SD 2.41) months of available wearable data during the 1-year prediagnostic window (13 months including the diagnosis month), corresponding to an overall missingness rate of 15.0%. Among cases, the median duration of available EHR history prior to diagnosis was 9.5 (IQR 4.7‐15.9) years.

**Table 1.** Baseline characteristics of adults with incident major depressive disorder and matched controls in a nested case-control study using linked electronic health record and wearable data from the All of Us Research Program (N=4104).

Characteristics	Overall	Case (n=829)	Control (n=3275)	SMD^a^
Age^b^ (y), median (IQR)	48.4 (36.3-61.3)	48.4 (36.2-61.2)	48.4 (36.3-61.4)	0.004
BMI^c^ (kg/m^2^), median (IQR)	29.9 (25.6-34.9)	29.8 (25.6-35.4)	29.9 (25.7-34.7)	0.046
Women, n (%)	3355 (81.7)	676 (81.5)	2679 (81.8)	0.033
Race, n (%)				0.098
Black or African American	271 (6.6)	40 (4.8)	231 (7.1)	
White	3384 (82.5)	691 (83.4)	2693 (82.2)	
Other^d^	449 (10.9)	98 (11.8)	351 (10.7)	
Ethnicity, n (%)				0.025
Hispanic or Latino	267 (6.5)	56 (6.8)	211 (6.4)	
Not Hispanic or Latino^e^	3837 (93.5)	773 (93.2)	3064 (93.6)	

a SMD: standardized mean difference. SMDs are presented as absolute values; values of less than 0.1 indicate negligible imbalance between groups.

b Age was defined at the diagnosis month for cases and the matched index month for controls.

c BMI was derived from measurements closest to 1 year before the diagnosis or matching month to minimize potential influence of emerging major depressive disorder on body weight.

d Participants with missing, unknown, or less frequently reported race categories were grouped into the “other” category in accordance with All of Us data reporting policies.

e Participants with missing or unknown ethnicity were grouped into the “Not Hispanic or Latino” category in accordance with All of Us data reporting policies.

### PA Trajectories Preceding Incident MDD Diagnosis

**Figure 2.**
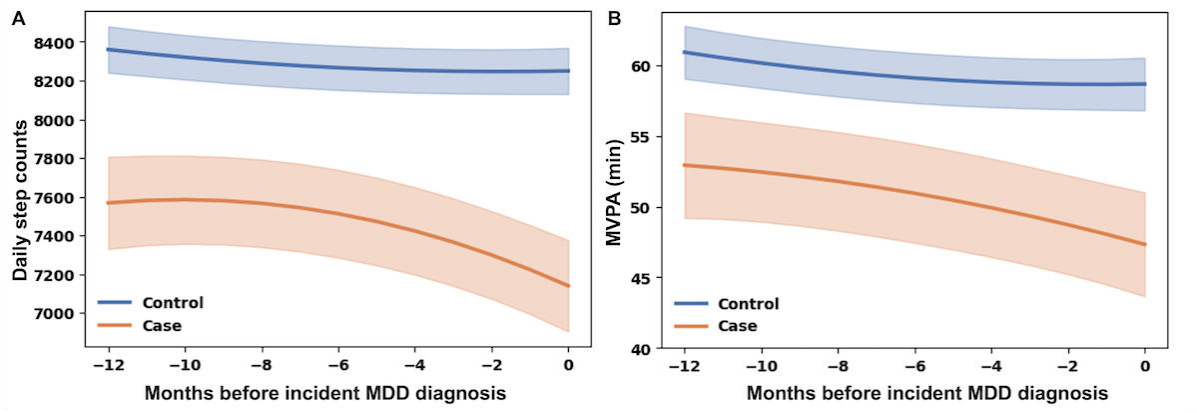
Prediagnostic trajectories of (A) daily step counts and (B) moderate to vigorous physical activity (MVPA) during 1 year before incident major depressive disorder (MDD) diagnosis in a nested case-control study from the All of Us Research Program**.** Lines represent model-estimated mean trajectories derived from linear mixed-effects models for cases and matched controls over time, with shaded areas indicating 95% CIs.

The trajectories of daily step counts during 1 year preceding the matching month differed significantly between incident MDD cases and matched controls (Figure 2A). In mixed-effects models, controls exhibited a relatively stable trajectory, whereas cases showed a marked and accelerating decline as the diagnosis month approached. These differences were supported by significant linear and quadratic interaction terms (time × case: β=−92.08, 95% CI −126.14 to −58.02, *P*<.001; time squared × case: β=−5.47, 95% CI −8.23 to −2.70, *P*<.001), with a significant global test for trajectory differences (joint Wald test: *P*<.001; Table 2). Model-derived marginal means indicated that daily step counts were consistently lower among cases than controls at every month examined. As early as 12 months before diagnosis, estimated daily step counts were 7568 (95% CI 7330‐7806) steps among cases, compared with 8360 (95% CI 8240‐8480) steps among controls (contrast difference: −792, 95% CI −1058 to −525 steps; *P*<.001). This case-control gap widened progressively over time, reaching −1109 (95% CI −1374 to −845; *P*<.001) steps in the matching month, with estimated daily step counts of 7140 (95% CI 6904‐7376) steps among cases and 8249 (95% CI 8130‐8369) steps among controls (Table S3 in Multimedia Appendix 1).

**Table 2.** Linear mixed-effects models comparing prediagnostic trajectories of daily step counts and moderate to vigorous physical activity (MVPA) between incident major depressive disorder cases and matched controls in the All of Us Research Program.

Term	β (95% CI)	*P* value	Global trajectory test, *P* value^a^
Daily step count model (unit: steps)			<.001
Intercept^b^	8249.57 (8130.24 to 8368.90)	<.001	
Group × case^c^	−1109.57 (−1373.76 to −845.39)	<.001	
Time^d^	3.66 (−11.58 to 18.90)	.64	
Time × case^e^	−92.08 (−126.14 to −58.02)	<.001	
Time squared	1.07 (−0.16 to 2.30)	.09	
Time squared × case^e^	−5.47 (−8.23 to −2.70)	<.001	
MVPA model (unit: min)			<.001
Intercept	58.69 (56.82 to 60.55)	<.001	
Group × case	−11.35 (−15.48 to −7.22)	<.001	
Time	0.05 (−0.23 to 0.32)	.75	
Time × case	−0.78 (−1.39 to −0.18)	.01	
Time squared	0.02 (−0.00 to 0.04)	.08	
Time squared × case	−0.04 (−0.09 to 0.01)	.10	

a The global trajectory test (joint Wald test) evaluated whether overall physical activity trajectories differed between cases and controls by jointly testing the interaction terms (time × case and time squared × case).

b The intercept represents the estimated mean physical activity level for the reference group (controls) at the reference time point (month 0).

c “Group × case” represents the baseline difference between incident major depressive disorder cases and matched controls (case-control status).

d Time was modeled on a retrospective monthly scale from −12 to 0 months relative to the diagnosis (or matching) month.

e “Time × case” and “time squared × case” represent differential linear and quadratic changes in trajectories between cases and controls over time.

Similar patterns were observed for daily MVPA trajectories (Figure 2B). Compared with controls, cases exhibited consistently lower daily MVPA levels throughout the prediagnostic period. In mixed-effects models, divergence in MVPA trajectories was supported by a significant linear interaction (time × case: β=−0.78, 95% CI −1.39 to −0.18, *P*=.01) and a significant global test for trajectory differences (joint Wald test: *P*<.001; Table 2). Model-derived marginal means showed that, at 12 months before diagnosis, estimated daily MVPA was 52.9 minutes among cases (95% CI 49.2‐56.7) and 60.9 minutes among controls (95% CI 59.1‐62.8) (contrast *P*<.001). This difference increased over time and reached −11.35 minutes (95% CI −15.48 to −7.22; *P*<.001) in the matching month, with estimated daily MVPA of 47.3 minutes among cases (95% CI 43.7‐51.0) and 58.7 minutes among controls (95% CI 56.8‐60.6; Table S4 in Multimedia Appendix 1).

### Within-Person PA Changes Before Diagnosis

To characterize the timing of within-person changes in PA preceding diagnosis, we conducted case-only marginal contrast analyses using 12 months before diagnosis (time=−12 months) as the reference. Among cases, daily step counts did not differ significantly from the reference level until 4 months before diagnosis. At −4 months, step counts were significantly lower than at −12 months (−145 steps, 95% CI −253 to −37; *P*=.02). Reductions became larger as diagnosis approached, reaching −428 steps in the matching month (95% CI −531 to −326; *P*<.001; Table S5 in Multimedia Appendix 1).

In terms of daily MVPA, levels among cases were significantly lower than the reference level by 5 months before diagnosis (time=−5 months; −2.48 minutes, 95% CI −4.32 to −0.64; *P*=.02). MVPA levels continued to decline over the prediagnostic period, reaching −5.61 minutes in the matching month (95% CI −7.35 to −3.86; *P*<.001; Table S6 in Multimedia Appendix 1).

### Subgroup Analyses

**Figure 3.**
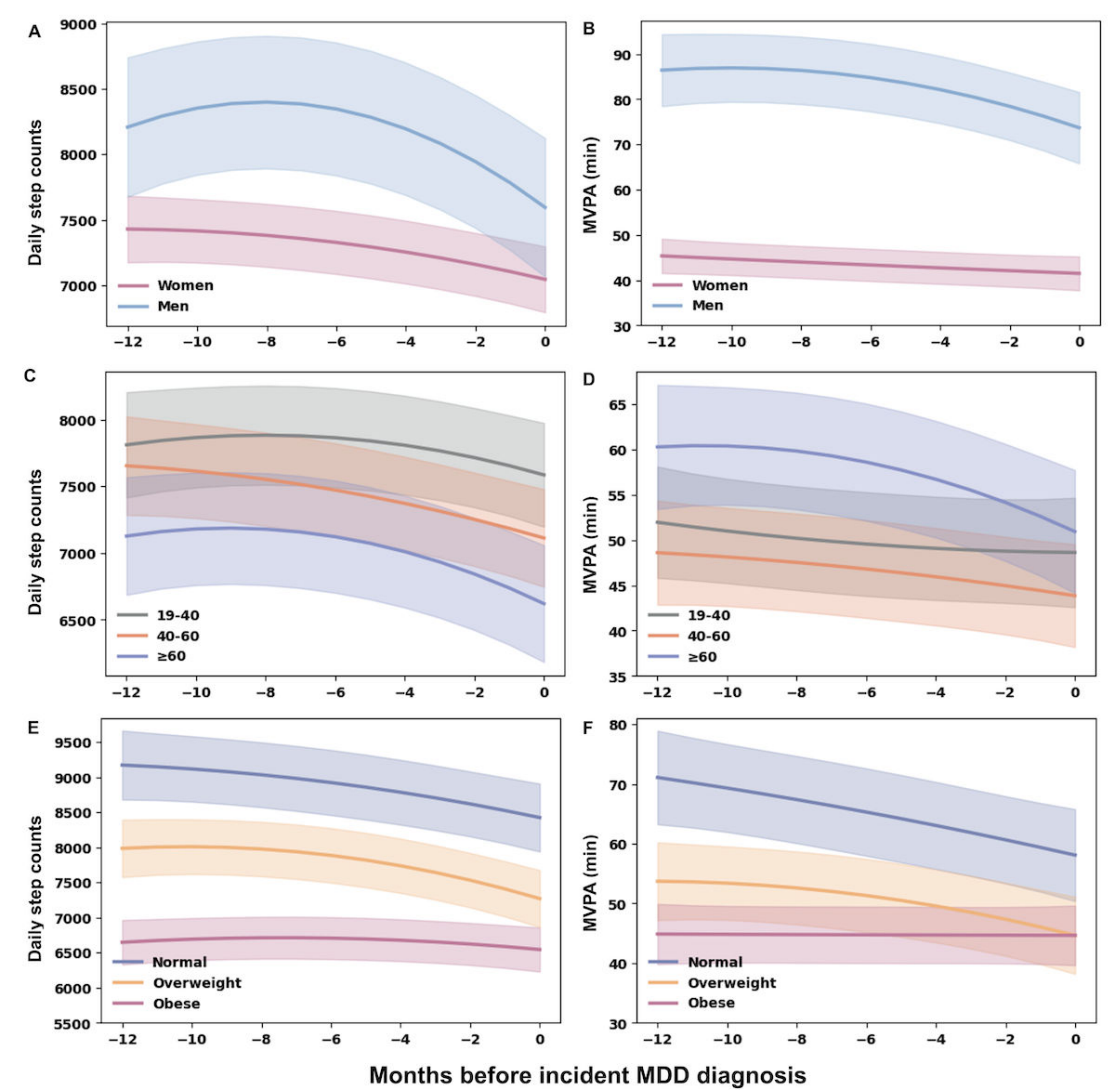
Subgroup-specific prediagnostic trajectories of physical activity during the 12 months before incident major depressive disorder (MDD) diagnosis in the All of Us Research Program. Panels show daily step counts and moderate to vigorous physical activity (MVPA) stratified by sex (A and B), age (C and D), and BMI (E and F). Lines represent model-estimated mean trajectories derived from linear mixed-effects models, with shaded areas indicating 95% CIs.

Case-only mixed-effects models revealed significant heterogeneity in prediagnostic PA trajectories by sex, age, and BMI (Figure 3; Tables S7 and S8 in Multimedia Appendix 1). Men exhibited steeper declines in both daily step counts and MVPA than women, supported by significant linear and quadratic time-by-sex interaction terms (joint Wald tests: *P*<.001).

PA trajectories also varied significantly by age and BMI category (joint Wald tests: *P*<.001). Participants aged 60 years and older exhibited lower overall daily step counts and greater reductions in daily MVPA over time. Across BMI categories, participants with overweight and obesity had lower overall levels of daily steps and MVPA than those with normal BMI, and participants with obesity showed comparatively attenuated temporal declines in both outcomes (Figures 3E and 3F). The underweight group was excluded from subgroup analyses because of insufficient sample size.

### Sensitivity Analyses

To assess whether device wear differed between cases and controls, we examined trajectories of monthly valid Fitbit wear days (Figure S1 in Multimedia Appendix 1). Wear days remained high and stable in both groups, with only a small between-group difference in average valid wear days per month (cases: 26.7, SD 5.3; controls: 27.0, SD 5.1 days per month). There was no evidence of a differential temporal decline in wear compliance (time × case: β=−0.007, 95% CI −0.032 to 0.018; *P*=.56).

Additional sensitivity analyses examined potential confounding from intercurrent medical conditions and variation in EHR history. Results were consistent with the primary analysis after excluding participants with newly recorded diagnoses of selected conditions that may affect PA within 1 year prior to diagnosis (n=713 in the restricted cohort; Table S9 in Multimedia Appendix 1). Similarly, results were consistent across strata of EHR history duration, including among participants with at least 5 and 10 years of EHR history prior to diagnosis (n=613 and n=319, respectively; Tables S10 and S11 in Multimedia Appendix 1).

## Discussion

### Principal Findings

In the AoURP cohort with linked wearable and EHR data, we found that adults who later received an incident MDD diagnosis exhibited a marked, progressively steepening decline in PA during the year preceding diagnosis compared with matched controls. Importantly, PA levels began to deviate from individuals’ usual patterns approximately 4 to 5 months before clinical diagnosis. This suggests a clinically relevant window in which persistent within-person downward trajectories, rather than transient drops, may represent a more informative signal of emerging change. These findings suggest that longitudinal, objective PA monitoring may provide clinically interpretable insights into functioning and psychomotor behavior, although careful evaluation of potential confounding and subgroup heterogeneity is warranted.

Our findings extend prior evidence linking lower PA to subsequent MDD diagnosis. Prior prospective cohort studies [10-12,17], including work in the AoURP cohort [33], have consistently associated lower PA levels with a higher risk of incident MDD. This pattern was also observed in our analysis, with cases exhibiting lower PA levels than matched controls throughout the year preceding diagnosis. However, most prior studies have relied on self-reported measures or infrequent accelerometer assessments, limiting their ability to capture continuous, long-term, objectively measured PA trajectories prior to clinical diagnosis. In contrast, by using longitudinal wearable-measured PA data, this study provides additional insight by characterizing both between-group differences and within-person changes over time. Specifically, we identified two key patterns: (1) cases showed significantly sustained declines in PA relative to controls, and (2) within cases, PA deviated from habitual levels approximately 4 to 5 months before diagnosis.

Prodromal depressive features [43-45] such as fatigue, anhedonia, and sleep disturbance may partially contribute to these observed prediagnostic PA declines, which may, in turn, exacerbate mood and sleep through behavioral and biological pathways, potentially contributing to a self-reinforcing cycle [11,46]. However, because EHR-documented diagnosis likely occurs after symptom onset and care seeking [8], these findings should be interpreted with caution. The observed declines in PA may reflect prodromal processes, early symptomatic impairment, or both rather than implying causal or temporal primacy of PA changes in MDD development.

Clinically, the primary implication of longitudinal, objective PA monitoring is not to diagnose MDD but to provide an early, scalable prediagnostic signal of emerging vulnerability in routine care. Such signals could help trigger depression screening, preventive strategies, or early intervention. Although no established threshold currently defines a clinically actionable decline in PA for incident MDD, there is evidence from other clinical populations suggesting that changes of approximately 500 steps per day may be clinically or practically meaningful [47,48]. In this context, the approximately 400-step decline observed in our study is modest but approaches this range. However, PA is inherently variable and influenced by a range of external and behavioral factors [49,50]. As such, fixed thresholds may be susceptible to transient fluctuations arising from nonpathological causes. Instead, our findings highlight a clinically relevant window in which sustained, within-person declines over several months, reflected in both the magnitude and slope of change, may provide a more informative signal of emerging vulnerability. Furthermore, PA is both a potentially modifiable risk factor for depression [6,51,52] and a well-established component of interventions that reduce depressive symptoms among individuals with established MDD [53,54]. Therefore, regardless of whether observed PA declines reflect prodromal vulnerability or early symptomatic impairment, timely prevention or intervention may confer clinical benefit [10,11,53-56].

We also observed substantial heterogeneity in prediagnostic PA trajectories by sex, age, and BMI. Male participants experienced steeper declines in both step counts and MVPA than female participants. Several factors may contribute to this pattern, including higher average activity levels among men [57,58], delayed help seeking for mental health symptoms [59,60], and sex differences in the types and social contexts of PA [61]. Age-related differences were also evident: while lower step counts in older adults are consistent with established age-related mobility patterns, more pronounced reductions in MVPA may reflect greater sensitivity of higher-intensity activity to emerging depressive symptoms, functional limitations, or comorbid conditions in later life [62,63]. In addition, persistently lower activity and flatter trajectories among individuals with obesity may reflect barriers such as weight-related functional limitations, pain, or reduced motivation [64]. These interpretations remain exploratory and warrant further investigation.

These findings highlight the potential utility of integrating consumer wearables into routine clinical and public health workflows. Continuous, passive monitoring of PA may enable scalable identification of individuals exhibiting sustained, within-person declines over time, which could serve as early behavioral signals of emerging vulnerability to MDD. Such signals could be incorporated into digital health platforms or clinical decision support systems to support risk stratification, enable targeted prevention, and facilitate earlier intervention prior to formal diagnosis. At a population level, these approaches could enable earlier identification of individuals at higher risk; support more efficient allocation of health care resources; and, ultimately, reduce the broader societal burden of MDD [65,66].

### Limitations

This study has several limitations. First, incident MDD was defined by the absence of EHR-documented diagnoses, consistent with prior AoURP methodologies [33,35,36], which may be influenced by the duration of available EHR history. Although sensitivity analyses restricted to longer EHR history yielded consistent results, left censoring and undiagnosed cases cannot be fully excluded. These are inherent limitations of EHR-based observational studies and warrant further investigation using complementary data sources and methods. Second, seasonality may influence both PA patterns and the timing of MDD onset [67]. In our study, cases and controls were matched on calendar time (matching month), which helps mitigate seasonal effects on between-group comparisons. However, seasonal influences on within-person trajectories cannot be fully excluded. Future studies incorporating more detailed environmental data may help further clarify these effects. Third, selection bias is also possible as inclusion required participants to have sufficiently long Fitbit monitoring histories and meet prespecified data completeness criteria, potentially limiting representativeness. Fourth, the study population was predominantly White, and the bring-your-own-device nature of Fitbit data contribution may have skewed the analytic sample toward more health-conscious or technologically engaged participants, potentially limiting generalizability. Fifth, missing data were handled under a missing at random assumption; however, in real-world wearable data, missingness may be influenced by unmeasured behavioral or contextual factors and, thus, may not fully satisfy this assumption. This represents a common challenge in mobile health studies and warrants further methodological investigation in future research.

### Conclusions

In this large cohort with linked wearable and EHR data, we characterized long-term, prediagnostic trajectories of objectively measured PA preceding incident MDD diagnosis. This addresses a key gap in prior research, which has largely lacked objective, longitudinal data on PA trajectories before clinical diagnosis. We identified sustained, within-person declines emerging 4 to 5 months before diagnosis, providing new insights into the temporal dynamics of behavior change prior to clinical recognition and defining a potential window for early detection. In real-world settings, these findings suggest that longitudinal monitoring of PA using consumer wearable devices may offer scalable and actionable signals to support risk stratification, targeted prevention, and earlier intervention for MDD. However, careful consideration of confounding and subgroup heterogeneity remains essential.

## Supplementary material

10.2196/93164 Multimedia Appendix 1 Supplementary figure and tables presenting Fitbit wear-time trajectories, diagnostic code definitions, additional model estimates, subgroup analyses, and sensitivity analyses.

## Abbreviations

AoURP: All of Us Research Program
EHR: electronic health record
MDD: major depressive disorder
MVPA: moderate to vigorous physical activity
PA: physical activity
