# Supplementary material for: Characterizing Physical Activity Trajectories Preceding Incident Major Depressive Disorder Diagnosis With Consumer Wearable Devices in the All of Us Research Program: Retrospective Nested Case-Control Study
Source: J Med Internet Res. 2026 May 4;28:e93164. doi: 10.2196/93164 (PMC13138713; doi:10.2196/93164)
Supplement: Multimedia Appendix 1 [file jmir-v28-e93164-s001.docx]

**Supplementary Figure 1. Trajectories of Monthly Valid Fitbit Wear Days in Cases and Controls**

**
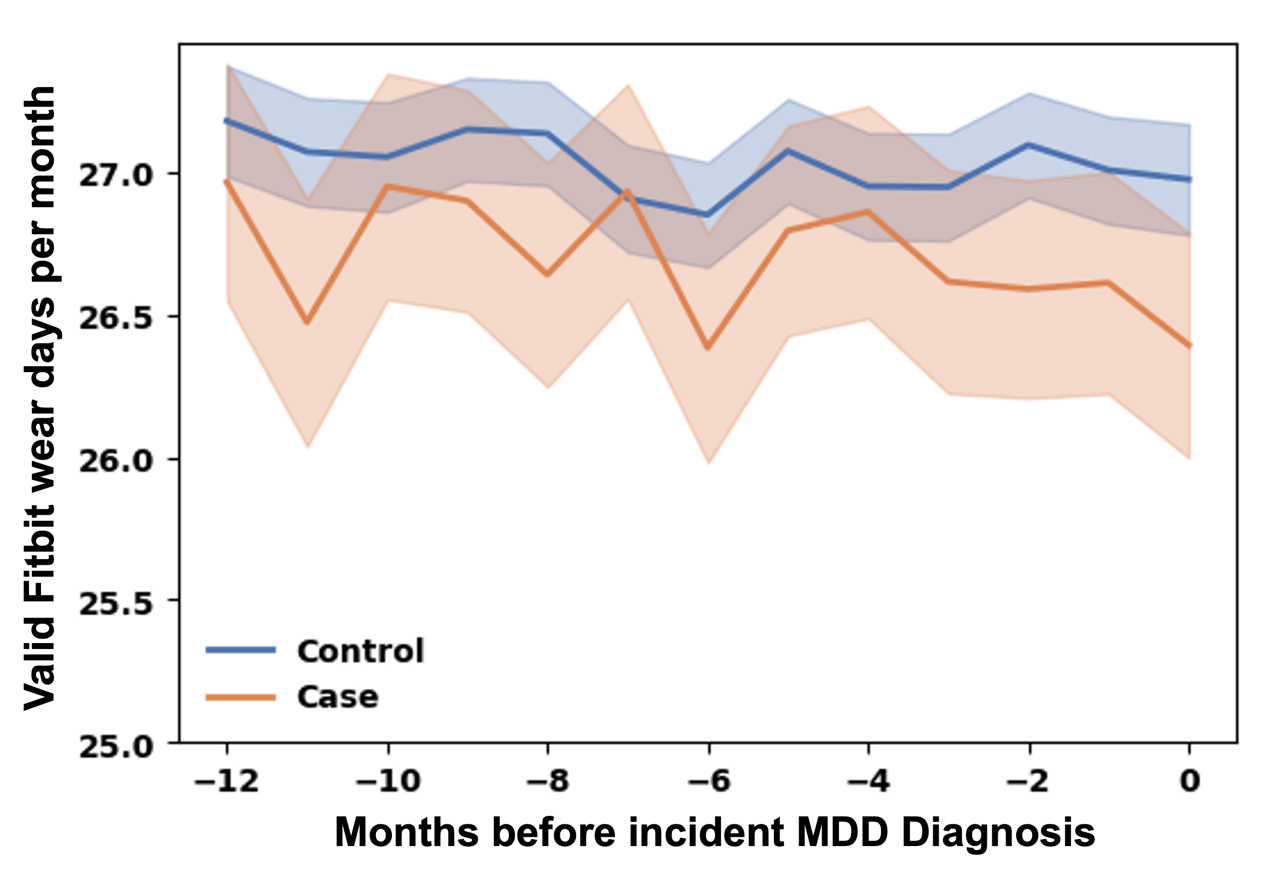
**

**Supplementary Table 1. OMOP Concept Identifiers and Corresponding ICD-9-CM and ICD-10-CM Codes Used for Inclusion and Exclusion.**

| **Category** | **Condition** | **OMOP concept ID** | **ICD-9-CM codes** | **ICD-10-CM codes** |
| --- | --- | --- | --- | --- |
| Inclusion | Major depressive disorder (single episode) | 4282096 | 296.2x | F32.x |
|  | Major depressive disorder (recurrent) | 4282316 | 296.3x | F33.x |
| Exclusion | Bipolar disorder | 436665 | 296.4x–296.8x | F31.x |
|  | Schizophrenia | 435783 | 295.x | F20.x |
|  | Schizoaffective disorder | 4286201 | 295.7x | F25.x |

**Supplementary Table 2. OMOP Concept Identifiers and SNOMED Codes for Medical Conditions Potentially Affecting Physical Activity Excluded in Sensitivity Analyses.**

| **Category** | **Condition** | **OMOP concept ID** | **SNOMED Code** |
| --- | --- | --- | --- |
| Musculoskeletal | Fracture of bone | 75053 | 125605004 |
| Cardiovascular | Myocardial infarction | 4329847 | 22298006 |
| Cardiovascular | Stroke (cerebrovascular accident) | 381316 | 230690007 |
| Cardiovascular | Transient ischemic attack | 373503 | 266257000 |
| Cardiovascular | Heart failure | 316139 | 84114007 |
| Cancer | Malignant neoplastic disease | 443392 | 363346000 |

**Supplementary Table 3. Model-Derived Marginal Mean Estimates of Daily Step Counts by Case–Control Status Across the Event-Aligned Timeline.**

| **Time (months)** | **Controls, steps (95% CI)** | **Cases, steps (95% CI)** | **Case–Control Difference^a^ (95% CI)** | **Contrast P^b^** |
| --- | --- | --- | --- | --- |
| −12 | 8360.14 (8240.32–8479.97) | 7568.21 (7330.16–7806.26) | −791.93 (−1058.44 to −525.43) | <0.001 |
| −11 | 8339.13 (8222.64–8455.61) | 7580.87 (7349.89–7811.84) | −758.26 (−1016.94 to −499.58) | <0.001 |
| −10 | 8320.25 (8205.37–8435.14) | 7584.73 (7357.22–7812.25) | −735.52 (−990.39 to −480.64) | <0.001 |
| −9 | 8303.53 (8189.14–8417.91) | 7579.81 (7353.46–7806.16) | −723.72 (−977.33 to −470.10) | <0.001 |
| −8 | 8288.95 (8174.51–8403.38) | 7566.10 (7339.74–7792.46) | −722.85 (−976.49 to −469.21) | <0.001 |
| −7 | 8276.51 (8161.89–8391.14) | 7543.60 (7316.91–7770.29) | −732.91 (−986.94 to −478.89) | <0.001 |
| −6 | 8266.23 (8151.50–8380.95) | 7512.31 (7285.47–7739.16) | −753.92 (−1008.12 to −499.71) | <0.001 |
| −5 | 8258.09 (8143.45–8372.72) | 7472.23 (7245.61–7698.85) | −785.85 (−1039.82 to −531.89) | <0.001 |
| −4 | 8252.09 (8137.67–8366.52) | 7423.36 (7197.21–7649.52) | −828.73 (−1082.18 to −575.27) | <0.001 |
| −3 | 8248.24 (8133.91–8362.57) | 7365.71 (7139.80–7591.61) | −882.54 (−1135.72 to −629.35) | <0.001 |
| −2 | 8246.54 (8131.80–8361.28) | 7299.26 (7072.61–7525.91) | −947.28 (−1201.32 to −693.24) | <0.001 |
| −1 | 8246.98 (8130.79–8363.18) | 7224.03 (6994.54–7453.51) | −1022.96 (−1280.18 to −765.74) | <0.001 |
| 0 | 8249.57 (8130.24–8368.90) | 7140.00 (6904.30–7375.70) | −1109.57 (−1373.76 to −845.39) | <0.001 |

^a^Difference calculated as marginal mean in cases minus marginal mean in controls.

^b^Contrast P-values were corrected using the Benjamini-Hochberg method.

**Supplementary Table 4. Model-Derived Marginal Mean Estimates of Moderate-to-Vigorous Physical Activity (MVPA) by Case–Control Status Across the Event-Aligned Timeline.**

| **Time (months)** | **Controls, minutes (95% CI)** | **Cases, minutes (95% CI)** | **Case–Control Difference^a^ (95% CI)** | **Contrast P^b^** |
| --- | --- | --- | --- | --- |
| −12 | 60.94 (59.06–62.81) | 52.94 (49.21–56.67) | −8.00 (−12.17 to −3.82) | <0.001 |
| −11 | 60.53 (58.73–62.34) | 52.72 (49.13–56.31) | −7.81 (−11.83 to −3.80) | <0.001 |
| −10 | 60.17 (58.40–61.95) | 52.46 (48.94–55.97) | −7.72 (−11.66 to −3.78) | <0.001 |
| −9 | 59.85 (58.09–61.61) | 52.15 (48.65–55.64) | −7.70 (−11.62 to −3.79) | <0.001 |
| −8 | 59.57 (57.80–61.33) | 51.79 (48.30–55.29) | −7.77 (−11.69 to −3.86) | <0.001 |
| −7 | 59.32 (57.55–61.09) | 51.39 (47.89–54.89) | −7.93 (−11.85 to −4.01) | <0.001 |
| −6 | 59.11 (57.34–60.88) | 50.95 (47.45–54.45) | −8.16 (−12.09 to −4.24) | <0.001 |
| −5 | 58.94 (57.18–60.71) | 50.46 (46.96–53.96) | −8.49 (−12.41 to −4.57) | <0.001 |
| −4 | 58.82 (57.05–60.58) | 49.92 (46.44–53.41) | −8.89 (−12.80 to −4.98) | <0.001 |
| −3 | 58.72 (56.96–60.49) | 49.34 (45.86–52.83) | −9.38 (−13.28 to −5.48) | <0.001 |
| −2 | 58.67 (56.90–60.44) | 48.72 (45.22–52.22) | −9.95 (−13.87 to −6.03) | <0.001 |
| −1 | 58.66 (56.86–60.46) | 48.05 (44.49–51.61) | −10.61 (−14.60 to −6.62) | <0.001 |
| 0 | 58.69 (56.82–60.55) | 47.33 (43.65–51.02) | −11.35 (−15.48 to −7.22) | <0.001 |

^a^Difference calculated as marginal mean in cases minus marginal mean in controls.

^b^Contrast P-values were corrected using the Benjamini-Hochberg method.

**Supplementary Table 5. Case-Only Contrasts of Daily Step Counts Relative to 12 Months Before Diagnosis.**

| **Month** | **Difference vs time = −12, steps (95% CI)** | **P value^a^** |
| --- | --- | --- |
| −12 | 0 (reference) | — |
| −11 | 12.59 (−16.52 to 41.71) | 0.595 |
| −10 | 16.41 (−37.08 to 69.90) | 0.73 |
| −9 | 11.44 (−61.73 to 84.62) | 0.828 |
| −8 | −2.30 (−90.62 to 86.02) | 0.959 |
| −7 | −24.82 (−123.83 to 74.18) | 0.748 |
| −6 | −56.13 (−161.68 to 49.43) | 0.51 |
| −5 | −96.21 (−204.46 to 12.04) | 0.163 |
| −4 | −145.07 (−252.74 to −37.40) | 0.02 |
| −3 | −202.71 (−307.47 to −97.96) | <0.001 |
| −2 | −269.14 (−370.28 to −167.99) | <0.001 |
| −1 | −344.34 (−443.65 to −245.03) | <0.001 |
| 0 | −428.32 (−530.91 to −325.73) | <0.001 |

^a^P-values were corrected using the Benjamini-Hochberg method.

**Supplementary Table 6. Case-Only Contrasts of Moderate-to-Vigorous Physical Activity (MVPA) Relative to 12 Months Before Diagnosis.**

| **Month** | **Difference vs time = −12, minutes (95% CI)** | **P value^a^** |
| --- | --- | --- |
| −12 | 0 (reference) | — |
| −11 | −0.22 (−0.71 to 0.27) | 0.387 |
| −10 | −0.48 (−1.39 to 0.43) | 0.325 |
| −9 | −0.79 (−2.04 to 0.45) | 0.255 |
| −8 | −1.15 (−2.65 to 0.36) | 0.18 |
| −7 | −1.55 (−3.23 to 0.14) | 0.108 |
| −6 | −1.99 (−3.79 to −0.19) | 0.051 |
| −5 | −2.48 (−4.32 to −0.64) | 0.017 |
| −4 | −3.01 (−4.85 to −1.18) | 0.003 |
| −3 | −3.59 (−5.38 to −1.81) | <0.001 |
| −2 | −4.22 (−5.94 to −2.50) | <0.001 |
| −1 | −4.89 (−6.58 to −3.20) | <0.001 |
| 0 | −5.61 (−7.35 to −3.86) | <0.001 |

^a^P-values were corrected using the Benjamini-Hochberg method.

**Supplementary Table 7. Subgroup Differences in Pre-Diagnostic Step-Count Trajectories Among Incident MDD Cases.**

| **Term** | **Coefficient (95% CI)** | **Term P value** | **Joint Wald test^a^** |
| --- | --- | --- | --- |
| Gender Model (ref: women) |  |  | <0.001 |
| group × men | 550.50 (-35.80 to 1136.79) | 0.07 |  |
| time^b^ | −62.09 (−96.17 to −28.01) | <0.001 |  |
| time × men | −137.55 (−215.46 to −59.64) | 0.001 |  |
| time² | −2.50 (−5.28 to 0.27) | 0.077 |  |
| time² × men | −9.87 (−16.18 to −3.57) | 0.002 |  |
| Age Model (ref: 19–40 years) |  |  | <0.001 |
| group × 40–60 | -472.51 (-1005.96 to 60.95) | 0.08 |  |
| group × ≥60 | -964.23 (-1549.36 to -379.09) | 0.001 |  |
| time | −73.95 (−127.37 to −20.53) | 0.007 |  |
| time × 40–60 | −0.56 (−73.16 to 72.04) | 0.99 |  |
| time × ≥60 | −50.97 (−129.53 to 27.59) | 0.2 |  |
| time² | −4.60 (−8.95 to −0.24) | 0.039 |  |
| time² × 40–60 | 2.15 (−3.76 to 8.05) | 0.48 |  |
| time² × ≥60 | −2.30 (−8.68 to 4.09) | 0.48 |  |
| BMI Model (ref: normal weight) |  |  | <0.001 |
| group × overweight | -1154.86 (-1786.43 to -523.28) | <0.001 |  |
| group × obese | -1880.42 (-2457.20 to -1303.64) | <0.001 |  |
| time | −104.01 (−171.54 to −36.48) | 0.003 |  |
| time × overweight | −41.69 (−129.04 to 45.67) | 0.35 |  |
| time × obese | 58.51 (−21.98 to 138.99) | 0.15 |  |
| time² | −3.47 (−8.99 to 2.04) | 0.22 |  |
| time² × overweight | −3.70 (−10.81 to 3.42) | 0.31 |  |
| time² × obese | 0.40 (−6.16 to 6.96) | 0.91 |  |

^a^Joint Wald tests evaluated whether linear and quadratic time effects differed across subgroups. All models were fitted within incident MDD cases only and included participant-specific random intercepts.

^b^Time was modeled on a retrospective scale from −12 to 0 months prior to diagnosis.

**Supplementary Table 8. Subgroup Differences in Pre-Diagnostic Trajectory of Moderate-to-Vigorous Physical Activity (MVPA) Among Incident MDD Cases.**

| **Term** | **Coefficient (95% CI)** | **Term P value** | **Joint Wald test^a^** |
| --- | --- | --- | --- |
| Gender Model (ref: women) |  |  | <0.001 |
| group × men | 32.23 (23.48 to 40.97) | <0.001 |  |
| time^b^ | −0.30 (−0.87 to 0.29) | 0.32 |  |
| time × men | −2.34 (−3.66 to −1.01) | 0.001 |  |
| time² | 0.00 (−0.05 to 0.05) | 0.92 |  |
| time² × men | −0.13 (−0.24 to −0.03) | 0.015 |  |
| Age Model (ref: 19–40 years) |  |  | <0.001 |
| group × 40–60 | -4.77 (-13.06 to 3.52) | 0.26 |  |
| group × ≥60 | 2.316 (-6.78 to 11.41) | 0.62 |  |
| time | −0.03 (−0.94 to 0.88) | 0.94 |  |
| time × 40–60 | −0.55 (−1.79 to 0.68) | 0.38 |  |
| time × ≥60 | −1.74 (−3.08 to −0.41) | 0.011 |  |
| time² | 0.02 (−0.05 to 0.10) | 0.59 |  |
| time² × 40–60 | −0.04 (−0.14 to 0.06) | 0.48 |  |
| time² × ≥60 | −0.10 (−0.21 to 0.01) | 0.061 |  |
| BMI Model (ref: normal weight) |  |  | <0.001 |
| group × overweight | -13.45 (-23.50 to -3.41) | 0.009 |  |
| group × obese | -13.45 (-22.63 to -4.28) | 0.004 |  |
| time | −1.32 (−2.47 to −0.17) | 0.025 |  |
| time × overweight | −0.15 (−1.64 to 1.34) | 0.84 |  |
| time × obese | 1.31 (−0.06 to 2.68) | 0.062 |  |
| time² | −0.02 (−0.11 to 0.07) | 0.68 |  |
| time² × overweight | −0.04 (−0.16 to 0.08) | 0.52 |  |
| time² × obese | 0.02 (−0.09 to 0.13) | 0.72 |  |

^a^Joint Wald tests evaluated whether linear and quadratic time effects differed across subgroups. All models were fitted within incident MDD cases only and included participant-specific random intercepts.

^b^Time was modeled on a retrospective scale from −12 to 0 months prior to diagnosis.

**Supplementary Table 9. Sensitivity Analysis Excluding Participants With Selected Medical Conditions^a^ Affecting Physical Activity Within 1 Year Prior to Diagnosis (n=713)**

| **Term** | **Estimate (95% CI)** | **Term *P* value** | **Global Trajectory Test (*P* Value)^b^** |
| --- | --- | --- | --- |
| Daily step count model (unit: steps)^c,d,e,f^ |  |  | <.001 |
| Intercept | 8067.39 (7941.25 to 8193.52) | <.001 |  |
| group × case | −845.88 (−1125.93 to −565.83) | <.001 |  |
| time | 3.07 (−13.53 to 19.66) | .72 |  |
| time × case | −97.64 (−134.87 to −60.41) | <.001 |  |
| time² | 1.14 (−0.20 to 2.48) | .09 |  |
| time² × case | −6.44 (−9.46 to −3.42) | <.001 |  |
| MVPA model (unit: minutes) |  |  | <.001 |
| Intercept | 56.35 (54.37 to 58.34) | <.001 |  |
| group × case | −8.48 (−12.89 to −4.07) | <.001 |  |
| time | 0.19 (−0.10 to 0.49) | .20 |  |
| time × case | −0.84 (−1.49 to −0.18) | .01 |  |
| time² | 0.03 (0.01 to 0.06) | .005 |  |
| time² × case | −0.06 (−0.11 to −0.00) | .04 |  |

^a^Medical conditions excluded in this analysis are listed in Supplementary Table 2.

^b^he global trajectory test (joint Wald test) evaluates whether overall physical activity trajectories differ between cases and controls by jointly testing the interaction terms (time × case and time² × case).

^c^Time was modeled on a retrospective monthly scale from −12 to 0 months relative to the diagnosis (or matching) month.

^d^The intercept represents the estimated mean physical activity level for the reference group (controls) at the reference time point (month 0).

^e^Group × case represents the baseline difference between incident major depressive disorder cases and matched controls (case–control status).

^f^Time × case and time² × case represent differential linear and quadratic changes trajectories between cases and controls over time.

**Supplementary Table 10. Sensitivity Analysis Restricted to Participants With ≥5 Years of EHR History Prior to Diagnosis (n=613).**

| **Term** | **Estimate (95% CI)** | **Term *P* value** | **Global Trajectory Test (*P* Value)^a^** |
| --- | --- | --- | --- |
| Daily step count model (unit: steps)^b,c,d,e^ |  |  | <.001 |
| Intercept | 8029.77 (7894.72 to 8164.82) | <.001 |  |
| group × case | −864.28 (−1164.02 to −564.53) | <.001 |  |
| time | −7.01 (−24.90 to 10.88) | .44 |  |
| time × case | −67.41 (−107.50 to −27.33) | .001 |  |
| time² | 0.13 (−1.32 to 1.57) | .86 |  |
| time² × case | −3.76 (−7.01 to −0.52) | .02 |  |
| MVPA model (unit: minutes) |  |  | <.001 |
| Intercept | 56.90 (54.80 to 59.00) | <.001 |  |
| group × case | −9.59 (−14.24 to −4.94) | <.001 |  |
| time | 0.46 (0.14 to 0.77) | .004 |  |
| time × case | −1.04 (−1.74 to −0.35) | .003 |  |
| time² | 0.06 (0.03 to 0.08) | <.001 |  |
| time² × case | −0.07 (−0.13 to −0.01) | .02 |  |

^a^The global trajectory test (joint Wald test) evaluates whether overall physical activity trajectories differ between cases and controls by jointly testing the interaction terms (time × case and time² × case).

^b^Time was modeled on a retrospective monthly scale from −12 to 0 months relative to the diagnosis (or matching) month.

^c^The intercept represents the estimated mean physical activity level for the reference group (controls) at the reference time point (month 0).

^d^Group × case represents the baseline difference between incident major depressive disorder cases and matched controls (case–control status).

^e^Time × case and time² × case represent differential linear and quadratic changes trajectories between cases and controls over time.

**Supplementary Table 11. Sensitivity Analysis Restricted to Participants With ≥10 Years of EHR History Prior to Diagnosis (n=391).**

| **Term** | **Estimate (95% CI)** | **Term *P* value** | **Global Trajectory Test (*P* Value)^a^** |
| --- | --- | --- | --- |
| Daily step count model (unit: steps)^b,c,d,e^ |  |  | <.001 |
| Intercept | 8130.19 (7953.93 to 8306.44) | <.001 |  |
| group × case | −974.43 (−1364.91 to −583.94) | <.001 |  |
| time | 13.77 (−8.92 to 36.45) | .23 |  |
| time × case | −96.73 (−146.89 to −46.57) | <.001 |  |
| time² | 1.61 (−0.22 to 3.44) | .09 |  |
| time² × case | −5.75 (−9.81 to −1.69) | .006 |  |
| MVPA model (unit: minutes) |  |  | <.001 |
| Intercept | 59.44 (56.59 to 62.30) | <.001 |  |
| group × case | −11.22 (−17.55 to −4.89) | .001 |  |
| time | 0.68 (0.27 to 1.08) | .001 |  |
| time × case | −1.17 (−2.07 to −0.27) | .01 |  |
| time² | 0.06 (0.03 to 0.09) | <.001 |  |
| time² × case | −0.07 (−0.14 to 0.01) | .07 |  |

^a^The global trajectory test (joint Wald test) evaluates whether overall physical activity trajectories differ between cases and controls by jointly testing the interaction terms (time × case and time² × case).

^b^Time was modeled on a retrospective monthly scale from −12 to 0 months relative to the diagnosis (or matching) month.

^c^The intercept represents the estimated mean physical activity level for the reference group (controls) at the reference time point (month 0).

^d^Group × case represents the baseline difference between incident major depressive disorder cases and matched controls (case–control status).

^e^Time × case and time² × case represent differential linear and quadratic changes trajectories between cases and controls over time.
